# The Assessment of *Proteus mirabilis* Susceptibility to Ceftazidime and Ciprofloxacin and the Impact of These Antibiotics at Subinhibitory Concentrations on *Proteus mirabilis* Biofilms

**DOI:** 10.1155/2013/930876

**Published:** 2013-09-12

**Authors:** Joanna Kwiecińska-Piróg, Krzysztof Skowron, Katarzyna Zniszczol, Eugenia Gospodarek

**Affiliations:** Department of Microbiology, Faculty of Pharmacy, Nicolaus Copernicus University in Toruń, Collegium Medicum of L. Rydygier in Bydgoszcz, M. Skłodowskiej-Curie 9, 85-094 Bydgoszcz, Poland

## Abstract

Rods of the *Proteus* genus are commonly isolated from patients, especially from the urinary tracts of the catheterised patients. The infections associated with biomaterials are crucial therapeutic obstacles, due to the bactericidal resistance of the biofilm. The aim of this study was to assess the susceptibility of *P. mirabilis* planktonic forms to ciprofloxacin and ceftazidime, the ability to form biofilm, and the impact of chosen sub-MIC concentrations of these antibiotics on biofilm at different stages of its formation. The research included 50 *P. mirabilis* strains isolated from wounds and the urinary tracts from patients of the University Hospital No. 1 in Bydgoszcz. The assessment of susceptibility to ciprofloxacin and ceftazidime was conducted using micromethods. The impact of sub-MIC concentrations of the chosen antibiotics on the biofilm was measured using the TTC method. The resistance to ciprofloxacin was confirmed for 20 strains (40.0%) while to ceftazidime for 32 (64.0%) of the tested *P. mirabilis* strains. All of the tested strains formed biofilm: 24.0% weakly, 26.0% moderately, and 50.0% strongly. It was determined that ciprofloxacin and ceftazidime caused eradication of the biofilm. Moreover, the connection between origin of the strains, biofilm maturity level, and resistance to antibiotics was proved.

## 1. Introduction

Bacteria from the *Proteus *genus are ciliated, Gram-negative rods, members of the Enterobacteriaceae family [1]. They were first characterised by Hauser in 1885 [2]. Currently, the genus *Proteus *consists of five species: *P. mirabilis, P. vulgaris, P. penneri, P. hauseri,* and *P. myxofaciens *and three genomic species of *P. genomospecies*: 4, 5, and 6 [1, 2].

*P. mirabilis *is the third most commonly isolated pathogen (after *Escherichia coli* and *Klebsiella pneumoniae*) of urinary tract infections [3, 4]. They are mostly ascending infections, more common among patients with anatomical or physiological malformations of the urinary tract, as well as among catheterised patients or due to medical care mistakes [3, 4].

Bacteria of this genus can cause infections of the respiratory system, wounds, bones, joints, digestive tract, and as well as, meningitis or bacteremia [5].

The therapeutic obstacles during *P. mirabilis* treatment can be connected with its ability to form biofilm [6, 7]. Biofilm is a formation of communicating microorganisms, adhering to certain surfaces and to neighboring cells, covered with an extracellular matrix [8]. It may consist of one or various species. Biofilm was first described in 17th century by Antonie van Leeuwenhoek, who observed bacteria from dental plates using an optical microscope [8, 9].

The ability to form biofilm promotes the development and persistence of infections connected with the use of biomaterials such as vascular and urinary catheters, ureteral or prostatic stents, penis and testicles implants, and heart valves or tracheal prosthesis [8, 10–12].

Biofilm-living bacteria exhibit different behaviour compared to their planktonic forms; moreover, they alter phenotypically [10, 13]. Different susceptibility to antibiotics of the biofilm forming cells compared to their planktonic forms is the main therapeutic problem. Antibiotic resistance of the biofilm can be caused by various coexisting mechanisms [13], such as mucus and glycocalyx, which reduce antibiotic distribution into the deeper layers of the biofilm [10]. These bacteria can also change their transcription and activate genes responsible for antibiotic resistance. Due to the nearness of cells, the transfer of genetic information is enhanced, even between different species or genera. This kind of information can be transferred *via* plasmids coding virulence factors and the mechanisms of antibiotic resistance. Furthermore, biofilm forming cells have the ability to communicate by the means of quorum sensing (QS) [9, 10, 13]. This enables the transferring of information connected with biocidal agents' resistance and the mechanisms of their activation [14].

The presence of antibiotics in the microorganisms' environment can additionally alter their genotype and phenotype [9, 10]. During antibiotic therapy, microorganisms are affected mostly by their concentrations being lower than the minimal inhibitory concentration (MIC), which is called subinhibitory concentration MIC (sub-MIC) [10]. The antibiotics at this concentration pose no lethal effect but can cause differentiation of the bacteria's surface and induce modifications of cellular functions like adhesion, hydrophobicity of the surface, and mobility of bacteria and also interfere with the interactions between host and bacteria, such as phagocytosis or reactive oxygen species production by the phagocytes [10, 13]. 

The aim of this study was the *in vitro *assessment of *P. mirabilis *planktonic forms' susceptibility to ceftazidime and ciprofloxacin, the determination of the ability to form biofilm among these strains, and the evaluation of the impact of chosen antibiotics on biofilm at different stages of its forming.

## 2. Materials and Methods

Fifty *P. mirabilis *strains were used in this study. They were isolated from urine (25; 50.0%) and wound swabs (25; 50.0%) and derived from 19 women (38.0%) and 31 men (62.0%) treated in the clinics of the Dr. Antoni Jurasz, University Hospital No. 1 in Bydgoszcz (SU1). Identification of the strains was conducted using one of the following tests: API 20E/ID32E (BioMerieux) and VITEK GN cards (BioMerieux) according to the manufacturers' recommendations. 

Strains were stored in a brain-heart infusion (BHI, Becton Dickinson) with 20.0% glycerol (POCH) at −70°C. For the current uses, strains were stored in cysteine-triptose agar (CTA; Becton Dickinson) for up to four weeks.

### 2.1. Assessment of MIC for Planktonic Forms

The assessment of the minimal inhibitory concentration (MIC) of ciprofloxacin (Sigma Aldrich) and ceftazidime (Sigma Aldrich) was conducted using the micromethod according to the EUCAST recommendations [15].

The ESBL resistance mechanism was determined with the disc diffusion method, using two discs, according to the National Reference Centre for Antimicrobial Susceptibility in Poland recommendations.

### 2.2. Biofilm Forming

Tested strains of *P. mirabilis *were propagated on the cystine lactose electrolyte deficient medium (CLED, Becton Dickinson) while the reference strains of *Staphylococcus aureus* 209P and *Escherichia coli* 35218 were obtained from American Type Culture Collection (ATCC), on 5.0% sheep blood agar (Becton Dickinson). Strains were cultured at 37°C for 18 hours. Next, the single colonies were inoculated into tryptic soy bullion (TSB, Bio-Rad) at 37°C. After 18 hours, cultures were centrifuged for 15 minutes at 4 000 rpm; then the supernatant was discarded and the pellet was rinsed with 3.0 mL of phosphate buffered saline solution (pH = 7.2) (PBS, POCH). Next, the bacterial suspension was centrifuged at 4 000 rpm for 10 minutes and the pellet was used to make the suspension of 0.5 MacFarland turbidity, using sterile Mueller-Hinton bouillon (MHB, Becton Dickson). Then, 20 *μ*L of every suspension was placed in the wells of polystyrene 96-well plate, in three repetitions. The wells were filled with 180 *μ*L of a sterile MHB medium, creating a 10-fold dilution. A sterility control was made of 200 *μ*L MHB medium in three repetitions. The culture was incubated in a humid chamber at 37°C for 24 hours. Then, the solutions were removed and the wells rinsed with sterile distilled water and left to dry at 37°C. Twenty minutes later, 200 *μ*L of methanol (POCH) was added to each well. The plates were placed on a shaker for 20 minutes at 400 rpm at room temperature. Next, the methanol was removed and the plates left to dry at 37°C for 20 minutes. In the next step, 200 *μ*L of 0.1% crystal violet (CV, POCH) were added to each well and placed in a shaker at 400 rpm for 10 minutes at room temperature. Next, the CV was removed by rinsing the wells with water thoroughly until the control wells became colorless. The plates were left for 20 minutes at 37°C for the water to evaporate. Finally, 200 *μ*L of methanol was added to each well and left on a shaker for 5 minutes at 400 rpm at room temperature.

**Figure 1:**
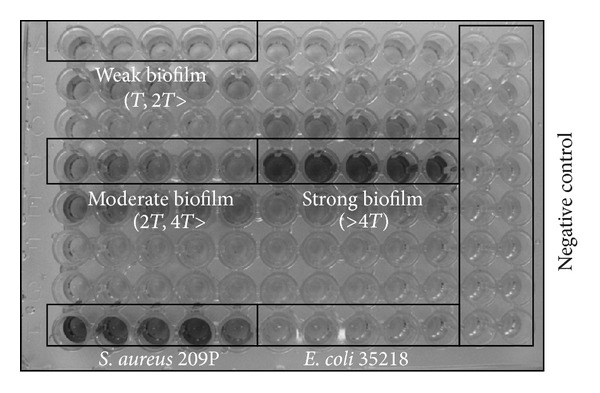
Visual diversity of *Proteus mirabilis* biofilm formation intensity.

Absorbance readings were conducted with a spectrophotometer at a wavelength of 570 nm, using KC4 v3.4 and KC4 Signature programs. To assess biofilm forming for each strain and negative control, the arithmetic mean of absorbance and standard deviation were used. The threshold value of absorbance (*T*) was proof of the biofilm forming and was defined as the sum of the arithmetic mean of negative control and a triple value of its standard deviation (*T* = *x*_nc_ + 3*δ*). A value below the calculated sum was recognized as, lack of biofilm. Mild biofilm was determined when the value of sum was between *T* and 2*T*, moderate biofilm—between 2*T* and 4*T*, and strong for a value higher than 4*T* (Figure 1). 

### 2.3. Assessment the Impact of Tested Antibiotics on *Proteus mirabilis* Biofilm

The 12- and 24-hour biofilms were formed according to the given methodology. After removing the medium containing the planktonic forms, 100 *μ*L of sterile MHB medium and 100 *μ*L of antibiotic were added to each well coated with the biofilm. The antibiotic concentrations were equivalent to 0.125, 0.25, 0.5, and 1.0 of MIC values, determined using three repetitions for planktonic forms of the tested strains.

The plates were placed in a humid chamber and incubated at 37°C. After 18 hours the cellular suspensions were removed and the biofilm rinsed three times with sterile distilled water. After that, the plates were left to dry for 20 minutes at 37°C. Next, 100 *μ*L of sterile TSB medium and 100 *μ*L of sterile 0.1% TTC solution (POCH) were added to each well. 

**Figure 2:**
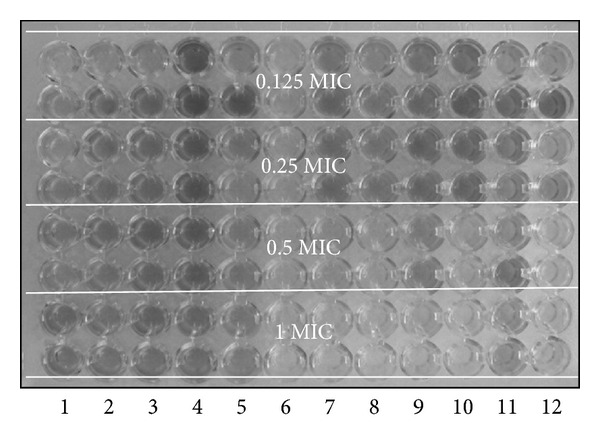
Evaluation of *Proteus mirabilis* biofilm eradication with examined antibiotics in selected sub-MIC concentrations.

The plates were incubated for two hours at 37°C. Then, the suspensions were removed and the plates rinsed three times. This was followed by the adding of 200 *μ*L of methanol to each well. Then, the plates were placed on shaker for 5 minutes at 400 rpm at room temperature. The read-outs were conducted with a spectrophotometer at 470 nm (Figure 2).

### 2.4. Statistical Analysis

Statistical analysis was conducted using the program *STATISTICA 10 ENG* (StatSoft Inc.). The normality of distribution was assessed. The significant differences between medians at *P* ≤ 0.05, which depended on the stage of biofilm forming, type of antibiotic, clinical samples origin, and the subinhibitory concentration of ciprofloxacin or ceftazidime and was determined according to the Kruskal-Wallis test. The detailed comparisons were conducted using the nonparametric Bonferroni's post hoc test.

## 3. Results

### 3.1. Antibiotic Susceptibility

Resistance to ceftazidime was determined for 32 (64.0%) while to ciprofloxacin for 20 (40.0%) of the tested *P. mirabilis* strains. The number of ciprofloxacin and ceftazidime resistant strains was higher among strains isolated from urine than those from wound swabs (Table 1).

Among the tested strains, 11 (22.0%) produced extended-spectrum beta-lactamases (ESBLs). From 11 of the *P. mirabilis* ESBL(+) strains, 7 (63.6%) were isolated from urine and 4 (36.7%) from wound swabs. Out of the 15 strains resistant to ciprofloxacin, the presence of ESBL was determined among 9 (60.0%) strains (Table 2). One of the ESBL(+) strains was ceftazidime susceptible.

The conducted research determined that 11, out of 50 *P. mirabilis* strains, were resistant either to ciprofloxacin or ceftazidime, and 16 to both antibiotics (Table 3). On the other hand, amongst strains resistant to ciprofloxacin, three were susceptible to ceftazidime, and among those ceftazidime-resistant strains, three were also ciprofloxacin susceptible. 

### 3.2. Biofilm Forming

**Figure 3:**
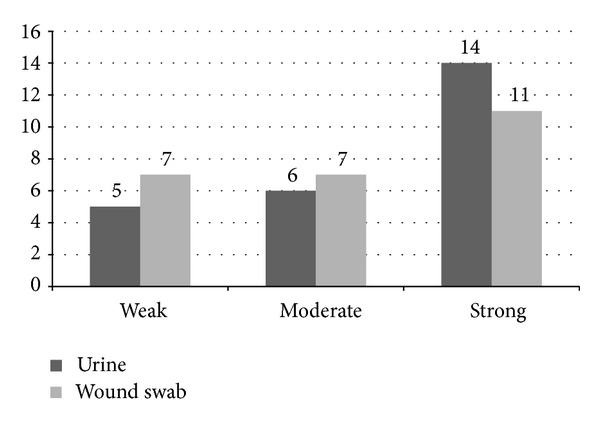
The intensity of *Proteus mirabilis *biofilm formation depending on the strains origin.

All tested *P. mirabilis* strains formed biofilm. A weak biofilm was formed by 12 (24.0%), moderate by 13 (26.0%), and strong by 25 (50.0%) of the tested strains (Figure 3). Strong biofilm forming was confirmed for 14 (56.0%) strains isolated from urine and 11 (44.0%) for strains isolated from wound swabs (Figure 3). No statistically significant differences (*P* > 0.05) of biofilm forming were determined in terms of the strains' origin.

Among the 32 strains susceptible to ciprofloxacin, 16 (50.0%) formed strong biofilm, and out of 20 ceftazidime-susceptible strains, 9 (45.0%) strains were found to form strong biofilm (Table 4).

### 3.3. The Impact of Subinhibitory Concentrations of Ciprofloxacin and Ceftazidime on 12- and 24-Hour *Proteus mirabilis* Biofilm

**Figure 4:**
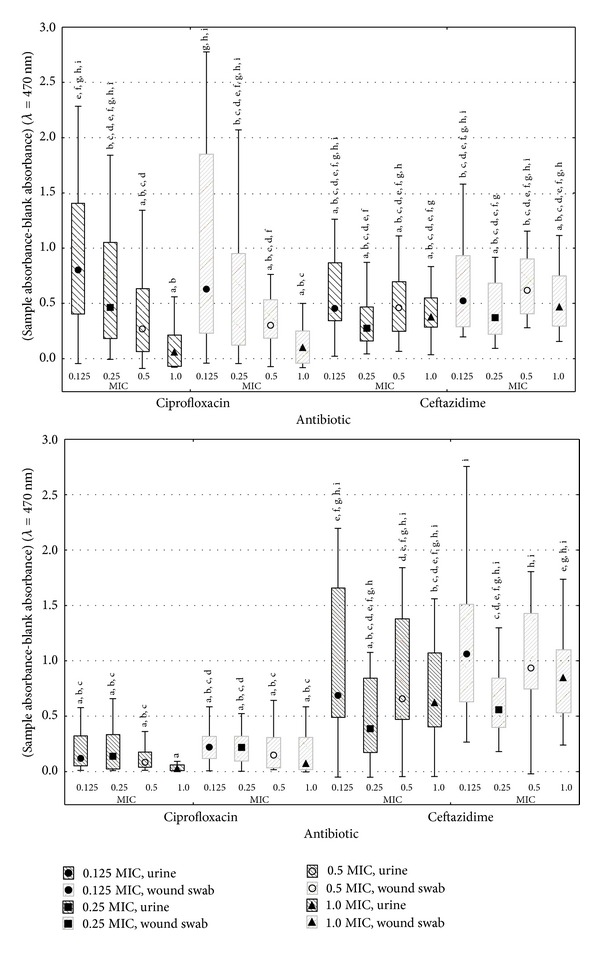
Effect of selected subinhibition concentrations of ciprofloxacin and ceftazidime on 12- and 24-hour *P. mirabilis* biofilm. a, b, c,…: statistically significant differences (*P* ≤ 0.05) between the elements marked with different letters (analysis covers both parts of diagram).

The obtained results led to the statement that both ciprofloxacin and ceftazidime eradicate *P. mirabilis* biofilm, which reflects the decrease of the absorbance median with increasing concentration of the antimicrobiological agent (Figure 4). The impact of biofilm maturity and the type of material, from which strains were isolated, were determined (Figure 4). The conducted research determined that both tested antibiotics varied in their influence.

The higher absorbance medians were stated (0.8029 and 0.4634 for strains from urine, 0.6292 and 0.3407 for strains from wound swabs) for ciprofloxacin than for ceftazidime (0.4548 and 0.2753, 0.5236 and 0.3703, resp.) using 0.125 and 0.250 sub-MICs to treat 12-hour biofilm (Figure 4). On the contrary, using 0.5 and 1.0 sub-MICs antibiotic values, the results were inverted. In both cases no statistically significant differences were determined (*P* > 0.05) (Figure 4).

In the case of 24-hour biofilm, ciprofloxacin was more effective in biofilm eradication than ceftazidime at all tested subinhibitory concentrations (Figure 4). For strains isolated from urine, the absorbance median varied from 0.0269 to 0.1384 relatively for 1.0 and 0.250 MIC, and for the strains isolated from wound swabs it varied from 0.0729 to 0.2206 at 1.0 and 0.128 MIC of ciprofloxacin (Figure 4). In case of ceftazidime, the absorbance medians were significantly higher and varied from 0.3873 to 0.6871 for strains isolated from urine and 0.5588 to 1.0616 for wound swabs-derived strains, correspondingly, at 0.250 and 0.128 MIC values (Figure 4). For both sources of isolates, a statistically significant higher effectiveness (*P* ≤ 0.05) of ciprofloxacin (compared to ceftazidime) against *P. mirabilis* biofilm in all concentrations was affirmed, excluding 0.250 MIC (Figure 4).

Regardless of the biofilm's maturity and the strains origin, the lowest absorbance medians were noticed at the concentration equal to 1.0 MIC for ciprofloxacin and 0.250 MIC for ceftazidime (Figure 4).

For 12-hour biofilm at 0.125 and 0.250 MIC ciprofloxacin concentration, lower absorbance medians were noticed in the cases of strains isolated from urine (absorbance medians were correspondingly 0.8029 and 0.4634) than in those of the strains isolated from wound swabs (absorbance medians were correspondingly 0.6292 and 0.3407) (Figure 4). In contrast, for the 0.5 and 1.0, the MIC results were inverted (Figure 4). No statistically significant differences were noticed (*P* > 0.05) (Figure 4). For 24-hour biofilm, lower absorbance medians, regardless of the concentration, were determined for strains isolated from urine, which proves higher susceptibility of the formed biofilm to ciprofloxacin, compared to the strains isolated from wound swabs (Figure 4). The differences were not statistically significant (*P* > 0.05) at the given concentration (Figure 4).

In case of ceftazidime, a higher susceptibility of biofilm was noted for strains isolated from urine, regardless of the biofilm's maturity and antibiotic concentration (Figure 4). No statistically significant differences were determined for given subinhibitory concentration (*P* > 0.05) (Figure 4).

 The use of ciprofloxacin caused significantly higher eradication of 24-hour biofilm compared to the 12-hour one, regardless of antibiotic concentration and sample origin (Figure 4). The determined absorbance medians for 12-hour biofilm treated with ciprofloxacin varied between 0.0589 and 0.8029 and for 24-hour biofilm between 0.0269 and 0.2206, depending on the sub-MIC value and sample origin (Figure 4). Statistically significant difference (*P* ≤ 0.05), caused by the biofilm's maturity, was determined only for the lowest ciprofloxacin concentration, regardless of the strains' origin.

Ceftazidime eradicated the 12-hour biofilm more efficiently than the 24-hour counterpart, which is reflected by the absorbance median values (0.2753–0.5236) noticed for the “younger” biofilm than by these of the 24-hour one (0.3873–1.0616) (Figure 4). At the given sub-inhibitory concentration for strains isolated from the same source, no statistically significant differences were determined (*P* > 0.05) in terms of the biofilm's maturity (Figure 4).

## 4. Discussion

According to the studies presented, 40.0% of the *P. mirabilis* strains were resistant to ciprofloxacin. This percentage is higher when compared to that of the results obtained by Hernández et al. [16], who indicated that 16.2% of strains are resistant to this fluoroquinolone. According to Ko et al. [17], only 13.6% of *P. mirabilis *strains were resistant to that antibiotic.

Kanayama et al. [18] found that among 46 ESBL(−) *P. mirabilis* strains, 23.9% were resistant to ciprofloxacin, while among ESBL(+), the corresponding value reached 89.3%. These results correspond with data obtained by Ho et al. [19], who determined that among ESBL(−) *P. mirabilis* strains, 14.0% exhibit resistance to ciprofloxacin, while the corresponding value for ESBL(+) strains reaches 76.9%. Moreover, Saito et al. [20] obtained similar results. From 80 of the ESBL(−) *P. mirabilis* strains, 13 (16.0%) were resistant to ciprofloxacin. The results of the current research confirm the trend mentioned above. Among ESBL(−) and ESBL(+) strains, accordingly 15.4% and 81.82% were resistant to ciprofloxacin. Compared to these numbers, a lower percentage of *P. mirabilis* resistant strains was obtained by Luzzaro et al. [21], which were 56.0% for ESBL(+) and 2.5% for ESBL(−). Both, own study and results of other authors, prove high contribution of strains resistant to ciprofloxacin among those producing extended spectrum beta-lactamase.

The studies presented determined that strains isolated from urine are more resistant to the ciprofloxacin than those from the wound swabs. These results are comparable to those of other authors' works. Guggenheim et al. [22] proved that 100% of wound swab-derived strains were susceptible to ciprofloxacin. Yah et al. [23] obtained a lower percentage (5.2%) of *P. mirabilis* strains from wound smears which were resistant to that antibiotic. According to Gales et al. [24], 81.5% of *P. mirabilis* strains isolated from urine were susceptible to ciprofloxacin. Saito et al. [20] showed that among 80 strains, 13 were resistant to the antibiotic mentioned above. On the other hand, Wagenlehner et al. [25] determined that 0 to 11.6% of *Proteus *spp. strains isolated from urine, between 1994 and 2000, exhibited resistance to ciprofloxacin.

In the current study 32 (64.0%) of *P. mirabilis* strains were resistant to ceftazidime. These results are comparable to those of Cao et al. [26], who determined that 99 (70.2%) strains were susceptible among 141 tested strains. A significantly higher contribution of susceptible strains was noticed by Ko et al. [17], Nijssen et al. [27], and Wang et al. [28]. The authors noted accordingly 90.9% [17], 95.3% [27], and 93.3% [28] ceftazidime-susceptible *Proteus *spp. strains. 

Cao et al. [26] indicated five (38.4%) ceftazidime-resistant strains out of 13 ESBL(+). Kanayama et al. [18] did not detect ceftazidime-resistant strains while testing 28 ESBL(+) and 46 ESBL(−) strains. These results differ from the results of this study, where 90.9% of ESBL(+) strains were resistant to the previously mentioned antibiotic. Among ESBL(−), 15.4% were resistant, 79.5% susceptible, and 5.1% intermediate.

The results of the study presented proved that strains isolated from urine were more resistant to ceftazidime than their wound swab-derived counterparts. The determined low contribution of ceftazidime-susceptible strains conflicts with Gales et al. results [24], which proved that among 74 of *P. mirabilis* strains isolated from urine in the years 1997–1999, 97.3% were susceptible to ceftazidime and in the year 2000, all 27 tested strains isolated from the same source were susceptible to the tested antibiotic. The results obtained by Lautenbach et al. [29] are similar; of *P. mirabilis* strains isolated from urine 91–100% were marked as ceftazidime susceptible. Wagenlehner et al. [25] proved that 0–4.5% of *Proteus *spp. strains isolated from urine were resistant to ceftazidime. Lockhart et al. [30] obtained similar results, according to which 5.2% of the strains isolated from urine were resistant. Moreover, Anguzu and Olila [31] determined a high percent (87.5%) of ceftazidime-susceptible *P. mirabilis* strains isolated from wound swabs. 

In the current study we also found that ceftazidime and ciprofloxacin sub-MIC had an impact on 12- and 24-hour biofilm formed by *P. mirabilis* at four antibiotic concentrations, corresponding to 0.125 MIC, 0.25 MIC, 0.5 MIC and 1 MIC values. The absorbance exhibited reverse correlation with the ciprofloxacin and ceftazidime concentration in all of the tested sub-MICs in both 12- and 24-hour biofilms.

Nucleo et al. [32] noticed that ESBL(+) *P. mirabilis* strains exhibited a greater ability to form biofilm within a wide range of its growth intensity, compared to the ESBL(−) strains. However, the study presented did not show such correlation. Both ESBL(+) and ESBL(−) formed biofilm at the comparable level.

Wasfi et al. [7] determined the inhibiting effect of the antibiotics against the biofilm. The influences of ciprofloxacin, ceftriaxone, nitrofurantoin and gentamycin sub-MICs were tested in the cases of adhesion of four *P. mirabilis* strains. It was proved that 0.5 MIC of all tested antibiotics reduces biofilm forming and that depletion accounted for 85.0% to 90.0%. Ciprofloxacin exhibited the highest reduction level: from 64.0% to 93.0% at 0.5 MIC and from 28.0% to 91.0% at 0.25 MIC [7].

Nucleo et al. [32] proved the inductive influence of antibiotics on biofilm forming capacity. They determined that an increase of imipenem and tazobactam concentrations leads to enhance the biofilm forming in all of the 10 tested *P. mirabilis* strains. The highest increase of biofilm forming was determined at 0.25 MIC for most of the strains, and only one exhibited the most intensive biofilm growth at 0.125 MIC.

In the current literature there is a lack of information on antibiotic impact on *P. mirabilis* biofilm at a different maturity stage. In the current study it was found that ciprofloxacin, regardless of its concentration and strain origin, eradicated the biofilm forming cells more efficiently in the case of 24-hour biofilm when compared to the 12-hour one. Ceftazidime exhibited higher biofilm eliminatory effectiveness for the 12-hour counterpart.

## 5. Conclusions

Knowledge of sub-MIC antibiotic concentration for microorganisms forming biofilm can be useful for rational antibiotic therapy. The presented results, and those of other authors, prove that different microorganisms exhibit diverse feedback to sub-inhibitory concentrations of various antibiotics. During antibiotic therapy, part of the microorganisms is affected by sub-inhibitory doses of drugs. Detailed knowledge of their impact on biofilm formed by various microorganisms, and also the pharmacodynamic indicators of medicines, can be useful at treating infections caused by the biofilm. Hence, further research to determine interactions between biofilm and biocidal agents is justified and necessary.

The conducted research proved that the efficiency of antibiotics against *P. mirabilis* biofilm depends on its maturity and strains' origin. Moreover, a concentration of ceftazidime significantly lower than the recommended MIC may be the most useful in the eradication of the biofilm. In most of the tested concentrations, ciprofloxacin was more efficient than ceftazidime against the *P. mirabilis* biofilm.

## Figures and Tables

**Table 1:** Susceptibility of *Proteus mirabilis* (*n* = 50) strains to ciprofloxacin and ceftazidime.

Antimicrobial susceptibility	Ciprofloxacin			Ceftazidime		
	Urine (*n* (%))	Wound swab (*n* (%))	Total (*n* (%))	Urine (*n* (%))	Wound swab (*n* (%))	Total (*n* (%))
Susceptible	9 (36.0%)	11 (44.0%)	20 (40.0%)	13 (52.0%)	19 (76.0%)	32 (64.0%)
Intermediate	8 (32.0%)	7 (28.0%)	15 (30.0%)	1 (4.0%)	1 (4.0%)	2 (4.0%)
Resistant	8 (32.0%)	7 (28.0%)	15 (30.0%)	11 (44.0%)	5 (20.0%)	16 (32.0%)

**Table 2:** Contribution of *Proteus mirabilis *strains producing beta-lactamase with extended spectrum of activity depending on the degree of susceptibility to examined antibiotics.

Antimicrobial susceptibility	Ciprofloxacin		Ceftazidime	
	ESBL(−) (*n* (%))	ESBL(+) (*n* (%))	ESBL(−) (*n* (%))	ESBL(+) (*n* (%))
Susceptible	20 (51.3%)	0	31 (79.5%)	1 (9.1%)
Intermediate	13 (33.3%)	2 (18.2%)	2 (5.1%)	0
Resistant	6 (15.4%)	9 (81.8%)	6 (15.4%)	10 (90.9%)

**Table 3:** The intensity of the *Proteus mirabilis* biofilm formation depending on the degree of susceptibility to the examined antibiotics.

	Ceftazidime			Total
	Susceptible	Intermediate	Resistant	
Ciprofloxacin				
Susceptible	16	1	3	20
Intermediate	13	0	2	15
Resistant	3	1	11	15

Total	32	2	16	50

**Table 4:** The intensity of the *Proteus mirabilis *biofilm formation depending on the degree of susceptibility to the examined antibiotics.

	Ciprofloxacin				Ceftazidime			
	S	I	R	Total	S	I	R	Total
The intensity of biofilm formation								
Weak	8	0	4	**12**	5	2	5	**12**
Moderate	8	0	5	**13**	6	4	3	**13**
Strong	16	2	7	**25**	9	9	7	**25**
Total	**32**	**2**	**16**	**50**	**20**	**15**	**15**	**50**

S: susceptible

I: intermediate

R: resistant.

## References

[1] O’Hara CM, Brenner FW, Miller JM (2000). Classification, identification, and clinical significance of *Proteus*, *Providencia*, and *Morganella*. *Clinical Microbiology Reviews*.

[2] Rózalski A, Kwil I, Torzewska A, Baranowska M, Staczek P (2007). *Proteus* bacilli: features and virulence factors. *Postępy Higieny i Medycyny Doświadczalnej*.

[3] Chen C-Y, Chen Y-H, Lu P-L, Lin W-R, Chen T-C, Lin C-Y (2012). *Proteus mirabilis* urinary tract infection and bacteremia: risk factors, clinical presentation, and outcomes. *Journal of Microbiology, Immunology and Infection*.

[4] Coker C, Poore CA, Li X, Mobley HLT (2000). Pathogenesis of *Proteus mirabilis* urinary tract infection. *Microbes and Infection*.

[5] Endimiani A, Luzzaro F, Brigante G (2005). *Proteus mirabilis* bloodstream infections: risk factors and treatment outcome related to the expression of extended-spectrum *β*-lactamases. *Antimicrobial Agents and Chemotherapy*.

[6] Jacobsen SM, Shirtliff ME (2011). *Proteus mirabilis* biofilms and catheter-associated urinary tract infections. *Virulence*.

[7] Wasfi R, Abd El-Rahman OA, Mansour LE, Hanora AS, Hashem AM, Ashour MS (2012). Antimicrobial activities against biofilm formed by *Proteus mirabilis* isolates from wound and urinary tract infections. *Indian Journal of Medical Microbiology*.

[8] Donlan RM (2001). Biofilm formation: a clinically relevant microbiological process. *Clinical Infectious Diseases*.

[9] Karatan E, Watnick P (2009). Signals, regulatory networks, and materials that build and break bacterial biofilms. *Microbiology and Molecular Biology Reviews*.

[10] Høiby N, Bjarnsholt T, Givskov M, Molin S, Ciofu O (2010). Antibiotic resistance of bacterial biofilms. *International Journal of Antimicrobial Agents*.

[11] Høiby N, Ciofu O, Johansen HK (2011). The clinical impact of bacterial biofilms. *International journal of oral science*.

[12] Whitfield H, Choong S (2000). Biofilms and their role in infections in urology. *BJU International*.

[13] Stewart PS (2002). Mechanisms of antibiotic resistance in bacterial biofilms. *International Journal of Medical Microbiology*.

[14] Braga PC, dal Sasso M, Sala MT (2000). Sub-MIC concentrations of cefodizime interfere with various factors affecting bacterial virulence. *Journal of Antimicrobial Chemotherapy*.

[16] Hernández JR, Martínez-Martínez L, Pascual A, Suárez AI, Perea EJ (2000). Trends in the susceptibilities of *Proteus mirabilis* isolates to quinolones. *Journal of Antimicrobial Chemotherapy*.

[17] Ko KS, Lee MY, Song J-H (2008). Prevalence and characterization of extended-spectrum *β*-lactamase-producing *Enterobacteriaceae* isolated in Korean hospitals. *Diagnostic Microbiology and Infectious Disease*.

[18] Kanayama A, Iyoda T, Matsuzaki K (2010). Rapidly spreading CTX-M-type *β*-lactamase-producing *Proteus mirabilis* in Japan. *International Journal of Antimicrobial Agents*.

[19] Ho PL, Ho AYM, Chow KH (2005). Occurrence and molecular analysis of extended-spectrum *β*-lactamase-producing *Proteus mirabilis* in Hong Kong, 1999–2002. *Journal of Antimicrobial Chemotherapy*.

[20] Saito R, Okugawa S, Kumita W (2007). Clinical epidemiology of ciprofloxacin-resistant *Proteus mirabilis* isolated from urine samples of hospitalised patients. *Clinical Microbiology and Infection*.

[21] Luzzaro F, Perilli M, Amicosante G (2001). Properties of multidrug-resistant, ESBL-producing *Proteus mirabilis* isolates and possible role of *β*-lactam/*β*-lactamase inhibitor combinations. *International Journal of Antimicrobial Agents*.

[22] Guggenheim M, Zbinden R, Handschin AE, Gohritz A, Altintas MA, Giovanoli P (2009). Changes in bacterial isolates from burn wounds and their antibiograms: a 20-year study (1986–2005). *Burns*.

[23] Yah SC, Eghafona NO, Oranusi S, Abouo AM (2007). Widespread plasmid resistance genes among *Proteus* species in diabetic wounds of patients in the Ahmadu Bello university teaching hospital (ABUTH) Zaria. *African Journal of Biotechnology*.

[24] Gales AC, Sader HS, Jones RN (2002). Urinary tract infection trends in Latin American hospitals: report from the SENTRY antimicrobial surveillance program (1997–2000). *Diagnostic Microbiology and Infectious Disease*.

[25] Wagenlehner FME, Niemetz A, Dalhoff A, Naber KG (2002). Spectrum and antibiotic resistance of uropathogens from hospitalized patients with urinary tract infections: 1994–2000. *International Journal of Antimicrobial Agents*.

[26] Cao V, Lambert T, Nhu DQ (2002). Distribution of extended-spectrum *β*-lactamases in clinical isolates of *Enterobacteriaceae* in Vietnam. *Antimicrobial Agents and Chemotherapy*.

[27] Nijssen S, Florijn A, Bonten MJM, Schmitz FJ, Verhoef J, Fluit AC (2004). Beta-lactam susceptibilities and prevalence of ESBL-producing isolates among more than 5000 European *Enterobacteriaceae* isolates. *International Journal of Antimicrobial Agents*.

[28] Wang H, Chen M, Ni Y (2010). Antimicrobial resistance among clinical isolates from the Chinese Meropenem Surveillance Study (CMSS), 2003–2008. *International Journal of Antimicrobial Agents*.

[29] Lautenbach E, Marsicano R, Heard M, Serrano S, Stieritz DD (2009). Epidemiology of antimicrobial resistance among gram-negative organisms recovered from patients in a multistate network of long-term care facilities. *Infection Control and Hospital Epidemiology*.

[30] Lockhart SR, Abramson MA, Beekmann SE (2007). Antimicrobial resistance among gram-negative bacilli causing infections in intensive care unit patients in the United States between 1993 and 2004. *Journal of Clinical Microbiology*.

[31] Anguzu JR, Olila D (2007). Drug sensitivity patterns of bacterial isolates from septic post-operative wounds in a regional referral hospital in Uganda. *African Health Sciences*.

[32] Nucleo E, Fugazza G, Migliavacca R (2010). Differences in biofilm formation and aggregative adherence between *β*-lactam susceptible and *β*-lactamases producing *P. mirabilis* clinical isolates. *New Microbiologica*.

